# Impact of Tung oil on a sustainable bio-based polymer, and development by zinc oxide nanoparticles

**DOI:** 10.1038/s41598-025-99556-x

**Published:** 2025-05-26

**Authors:** Hamdy M. Naguib

**Affiliations:** Department of Petroleum Applications, Egyptian Petroleum Research Institute (EPRI), Nasr City, Cairo, 11727 Egypt

**Keywords:** Tung oil, Polyester, Solid waste, Zinc oxide nanoparticles, Environmental sciences, Chemistry, Engineering, Materials science

## Abstract

The use of natural and bio-based materials instead of petrochemicals is strongly recommended for reducing greenhouse gas emissions. Here we aim to promote the environmentally friendly bio-based polyester (P), prepared from biomass, with natural Tung oil (TO) plasticizer and zinc oxide nanoparticles (ZnO NPs) filler by 10–50%, and 3% to get a sustainable nanocomposite. The grafting altered the profile of neat P. Owing to insufficient contents, low concentrations have a slight impact, and high concentrations have more enhancements. The physical properties accompanied by curing of P/TO copolymer showed a decrease in viscosity, gelation time, and gelation-curing period for TO-based specimens, besides the lower heat emission during curing reaction, compared with that of P, by 3.4% and 4%, respectively, for P/TO-40 and P/TO-50 copolymers. The stability against exudation was promoted by 48.6%, where all concentrations of composites are more stable than P. P/TO-40 and P/TO-50 improved creep resistance by 62% and 88.1%, respectively, due to the stable surfaces. Furthermore, P/TO-50 concentration reduced hardness by 25%, but it was improved by ZnO NPs by 46.7%. Both TO plasticizer and nanofiller make the polymer capable of absorbing the flexural loading as a toughened composite. The proposed composites provide positive effects on the thermal behavior. Particularly, the P/TO-50 formula decreased the value of tan Delta by 35.5%; all composites increased T_*g*_ as well. The obtained data-results and SEM photos confirm the grafting and good distribution of TO plasticizer and ZnO NPs into P matrix through an improved and stable homogeneous bio-based polymer nanocomposite.

## Introduction

Recently, the use of natural products and environmentally friendly materials to reinforce polymers has been encouraged to achieve the goals of sustainable development. It is well known that polyester is considered one of the most commonly applied polymers; some of its applications are construction laminations, printed products, coating, cast forms and blocks, etc^1,2^. In spite of its good characteristics, polyester suffers from brittleness, moderated thermal stability, low bending properties, and other related disadvantages^3^. With incorporation of novel reinforcements and fillers, the characteristics of traditional materials may be altered. Consequentially, reinforcements are included in the conventional materials to enhance their diverse qualities^4^. For the plasticizing filler, its effectiveness was found to change the mechanical properties of the polymer matrix^5^. Because of the industrial source of these fillers, the applications of their composites are limited due to lack of sustainability, and harmful effects on the environment. Different recycled materials, like solid waste, recycled and low-carbon materials, can be used and exploited as additives/fillers for preparing sustainable and low-carbon composite products^6–8^. Other polymers are subjected to recycling, and compounded within other reactants for production of new low-cost products^9,10^. Bio-based polyesters represent a promising area of research and development, driven by the need for sustainable materials. Their production involves various synthetic strategies that yield polymers with diverse properties suitable for a wide range of applications. Continued advancements in the synthesis and modification of these materials are essential for overcoming existing limitations and enhancing their commercial viability. Bio-based polyesters are a class of polymers synthesized from renewable resources, such as plant-based materials and microorganisms, which offer a sustainable alternative to petroleum-derived polyesters. The primary structures of bio-based polyesters typically consist of ester linkages formed through the polymerization of bio-derived diacids and diols. Common examples include polylactic acid and polybutylene succinate, which are derived from lactic acid and succinic acid, respectively. These materials are characterized by their biodegradability, biocompatibility, and reduced toxicity, making them suitable for various applications. The structural characteristics of bio-based polyesters play a crucial role in determining their properties^11–13^. The production of bio-based polyesters generally involves two main synthetic routes: polycondensation and polymerization. In the first, diols and diacids react to form polyesters while releasing water. The polymerization allows for the tuning of molecular weight and crystallinity, which significantly affects the thermal and mechanical properties of the cured polymer^14,15^.

The trend of utilizing ecofriendly additives is increasing, especially natural products^16–18^. Tung oil, as a natural oil type, is a triglyceride material extracted from Tung trees. Mainly, it contains unsaturated acids, such as eleostearic, oleic, and linoleic acids; such nature facilitates the compounding with polymers throughout functional sites^19,20^. Some trials were reported focusing on utilization of Tung oil for plasticization and controlling the properties of different polymeric matrices. Unsaturated polyester-dicyclopentadiene polymer was modified with Tung oil by melting polycondensation and then blended with styrene comonomer to give a cured and tough polymer. The impact and tensile properties were changed due to the Tung oil-based polymer as an alternative to petroleum-based additives^21^. Tung oil with chitosan, as second filler, developed epoxy resin; the mechanical and thermal properties of the polymer were adjusted through crosslinking among all contents^22^. Another epoxy type was toughened by cyano-treated Tung oil. Compared with epoxy, the investigations on thermal, surface, and mechanical properties conducted on cured samples showed a significant improvement, compared with industrial types^23^. For a green plasticizer, vegetable oil modifiers allowed for varying the properties of Tung oil networks obtained by polymerization to increase the efficiency of Tung oil. The proposed treatment provided a good damping effect in different conditions^24^. A new polymer-modified Tung oil waterborne varnish was developed. The green product improved the thermal and mechanical stability and insulation, and decreased water adsorption, in addition to the waterborne ability^25^. A Tung oil-maleic triglycidyl ester was synthesized and included in polyvinyl chloride as a replacement for dioctyl phthalate plasticizer; the interaction between the polymer and the recommended modified ester was attempted^26^.

Regarding the overall superior profile, it was reported that nanoparticles of numerous metal oxides play a vigorous role in endorsing the thermal behavior of materials in addition to their mechanical, catalytic, electrical, isolative, physical, and other characteristics. Of them, the environmental zinc oxide nanofiller is an effective type^27–32^. In addition, nanoparticles of green carbonates have been applied to promote the thermal and mechanical properties of low-profile polymers^33,34^. ZnO and ZnS were adsorbed and utilized as a potential fire-retardant coating on fabrics. The hybrid material was found to alter factors such as heat release rate, smoke emission, and mass loss rate^32^. In a related study, ZnO NPs were precipitated on fabric surfaces along with a type of phosphonopropionamide. Different modes of analysis confirmed the deposition of zinc and phosphorus components, in addition to the fire-retardancy impact^35^. Another coating material, as transparent layers comprising ZnO NPs and organophosphate, was fabricated. As well as the smoke suppression, fire and aging protection, and antimicrobial performance, ZnO improved the mechanical properties and transparency profile^36^. An organometallic composite, containing zinc, iron, phosphorus, and aminopyridine, was formulated on epoxy polymer. The dissociation of polymers into more aromatic structures could form dense carbon shielding layers^37^. Another thermosetting epoxy coating was grafted by a dual system of precipitated ZnO NPs/charring foaming agent, altering the thermal decomposition, increasing char formation, and minimizing oxygen flow^38^. New nanocomposites, containing chitosan-modified ZnO NPs and aromatic polyamide, were designed by solution casting for filling polyvinyl chloride. Both additives stabilized the matrix, and increased the tensile strength and mass-loss temperature^39^. The influence of polysiloxane-treated ZnO NPs on polypropylene was studied. Compared with the neat matrix, the 16 wt% loading percentage succeeded in passing the UL 94v-0 standard and increasing the limiting oxygen index and tensile strength. Also, the polymer kept a smooth surface with accepted mechanical properties after UV irradiation^40^. In another thermoplastic polymer, hybrid nanofillers based on cellulose nanocrystals and ZnO NPs were investigated for promoting extruded polylactic acid regarding mechanical and thermal characteristics. The combination of 1.5% enhanced the mechanical and thermal characteristics, and increased char formation. However, the sole ZnO NPs hastened the thermal degradation of polylactic acid, and decreased its modulus value due to incompatibility^41^. ZnO NPs and phosphazene-triazine bigroup were doped, and added to polylactic acid; although the decline occurred in the mechanical properties, the thermal effect was promoted because of the decomposition to low molecular segments, and char-forming molecules^42^. This study aims to prepare and characterize a novel environmentally friendly nanocomposite using bio-based polyester, natural Tung oil, and zinc oxide nanoparticles. The synergetic effect of both additives on the physical, mechanical, and thermal characteristics is studied in details to enhance the bio-based polyester. Related to the aspects of UNSDGs, in particular the SDGs 12 and 13 that belong to sustainable consumption and production and climate change, the proposed sustainable composites target these goals regarding preserving and recycling natural resources and waste, reducing climate change, innovation, and promoting economic growth. Modifying the bio-based polyester with these materials is a possible utilization of sustainable products compared with petroleum-based polymer.

## Experimental and methods

### Materials and preparations

**Fig. 1:**
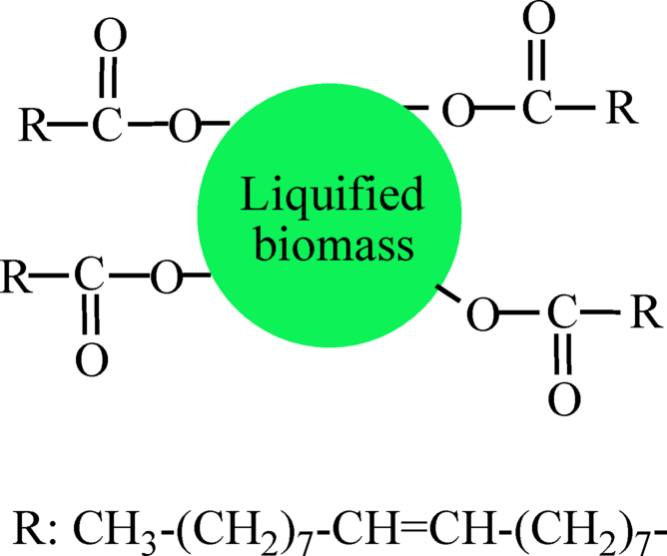
Structure of the bio-based polyester (P) matrix.

The materials used in this paper are indicated here. Tung oil (TO) plasticizing derivative type, obtained from Shandong-Deshang Chemical Ltd. with a viscosity of 203 cP, was taken as the green plasticizer additive. Zinc oxide nanoparticles (ZnO NPs) were used as the nanofiller modifier; they were obtained from Alfa Aesar Chemicals with an average particle size of ~ 70 nm. The polymer needed to be modified is the bio-based polyester (P) that was prepared from biomass waste^43^. Briefly, a biomass mixture waste was subjected to liquification process, and then was reacted with oleic acid to get this bio-based polyester. The structure of P matrix is given in Fig. 1. Methyl ethyl ketone peroxide type, purchased from Singapore Highpolymer, was the catalytic agent.

Experimentally, the modification of P was accomplished as indicated in the following steps: The bio-based P matrix was modified by TO plasticizer with different concentrations between 10% and 50% to produce P/TO-10, P/TO-20, P/TO-30, P/TO-40, and P/TO-50 copolymers. As per the low cost and availability of TO plasticizer, there will be a reduction of the total cost by about 18% in case of consuming 50% TO. Grafting TO onto the polyester backbone was performed by solution mixing. Both liquid phases were mixed with the help of stirring and ultrasonication. The produced copolymer is composed of a mixture of unsaturated polyester, which originated from liquefied biomass and oleic acid, and unsaturated Tung oil plasticizer. ZnO NPs were also added by 3% to promote some characteristics, as given in the discussion section. ZnO NPs were incorporated into the P/TO matrix by solution mixing. The viscosity of P/TO-50/ZnO NPs system reads 330 cP, which is close to that of P/TO-50, after mechanical mixing and ultrasonication. In the related specimens, TO and ZnO NPs filler were added to the polyester matrix. In an ice bath, ultrasonic waves were applied for mixing, and then mechanical stirring was adjusted for about 20 min. The application of ultrasonic waves and further mechanical stirring causes a good distribution of fillers in the matrix, as presented later by SEM photos. The crosslinking of proposed composites was promoted by the gentle mixing of 1% methyl ethyl ketone peroxide with the prepared formulas for one minute at room temperature. The whole system was mixed gently to let al.l ingredients contact each other completely. Finally, the composites were kept overnight at room temperature, then heated to 65 °C for 2 h for completion of crosslinking reaction^44,45^.

### Investigations

The following investigations were conducted to analyze the prepared copolymers and composite. The viscosity of polymers was identified by the Brookfield-DV2 T digital viscometer following ASTM D-789. The polymer reactivity, including gelation time, curing temperature, and gelation-curing period, was identified following ASTM D2471. The polymer specimens were poured in cups; after adding the curing agent, the gelation phase was checked by a glass rod where the polymer starts to stick with this rod. Both gelation time and gelation-curing period are calculated by a stopwatch. The stability of composites to exudation was measured by calculating the exudation percentage as the weight loss after laying the specimen between couples of filter papers at 50 °C for 24 h. The underlying mechanism for the improved stability originates from the low exudation percentage, where the specimen doesn’t exude more of its contents. The lower the exudation percentage recorded, the more improved stability is for the specimen; the exudation percentage is expressed using Eq. 1^26^, where m and m` denote respectively the initial weight and the weight after exposing the specimen to exudation conditions.

1 $$\hbox{Exudation percentage}\ (\%) = (m - m`)/m \times 100$$

The morphology was detected by a scanning electron microscope (FEI Nova NanoSEM 450) using the backscattered electrons SEM imaging with a 5 kV accelerating voltage. The taken specimens were coated with gold before capture. The WANCE creep rupture machine was used to investigate the creep resistance of composites, following ASTM D 2990. Specimens with dimensions of 3 × 10 cm^2^ were fixed on the testing plate in the tensile mode at room temperature. The testing was executed at a stress of 10 N for determining the creep response. The Barcol impression test was conducted to identify the hardness of P and its proposed products following ASTM D 2583. The Barcol hardness is measured through indentation of the impressor’s needle into the specimen; the penetration indenter reads the hardness value. The type of Gardco Barcol impressor was used by applying a constant manual impression to 5 × 5 cm^2^ specimens. The effect of environmental modifiers on the flexural strength was studied by the XLC H-universal testing machine according to ASTM D 790. About 1.3 × 12.7 cm^2^ specimens were subjected to increased flexural load till failure. The bending test was performed by 3-point bending mode, with measuring the flexural strength parameter. The thermal analysis of composites was evaluated by the dynamical mechanical analyzer (DMA) using the NEXTA machine following the standard of ASTM D 4065. The corresponding glass transition temperature and damping peak parameters were identified. Specimens with dimensions of 1 × 5 cm^2^ were heated gradually from room temperature to 120 °C under flexural force for detecting the related thermal properties. The response surface methodology (RSM) statistical analysis was projected by the Box-Behnken designing to study the significance and effect of the presented treatmet factors on the resulting responses.

## Results and discussion

### Curing of copolyester

**Fig. 2:**
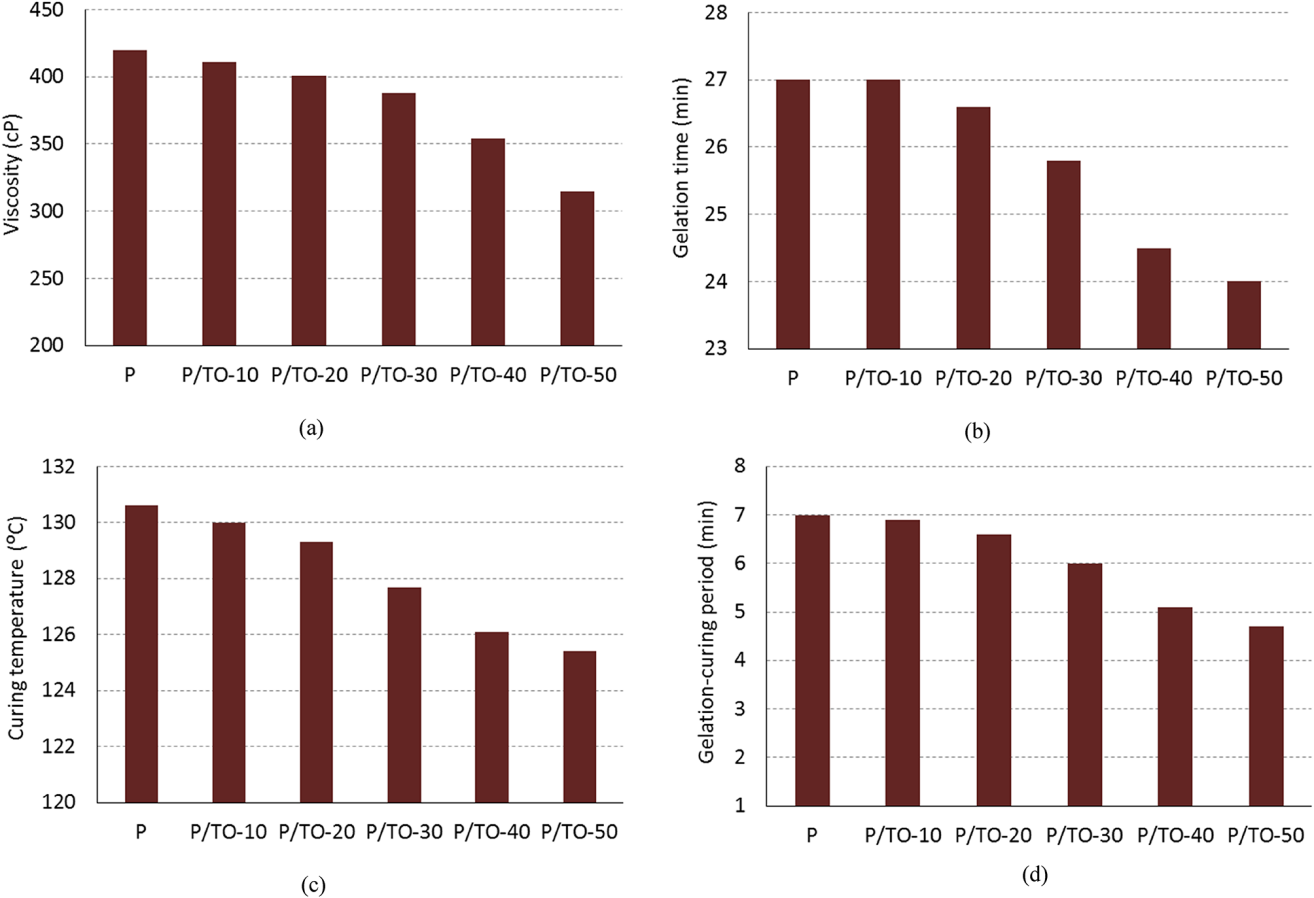
Curing properties of bio-based polyester, and Tung oil-based copolymers: Viscosity (a), Gelation time (b), Curing temperature (c), and Gelation-curing period (d).

The physical properties accompanied with the curing of the copolyester are studied here. Respectively, Figs. 2(a), (b), (c), and (d) illustrate the viscosity, gelation time, curing temperature, and gelation-curing period for the bio-based polyester, and its Tung oil-based copolymer. As per Fig. 2(a), the viscosity of the proposed polyester is altered. Different polymers become less viscous when plasticizers or toughening materials are incorporated due to the easier slipping of polymer layers on each other^46,47^. Actually, TO has a viscosity of 203 cP, which is lower than that of polyester (420 cP). So, the overall viscosity reduced after mixing. This is noticed in the viscosity measured for P/TO-10, P/TO-20, P/TO-30, P/TO-40, and P/TO-50 that gave 411, 401, 388, 354, and 315 cP, respectively, compared with 420 cP of neat P. There are two points to be demonstrated: Firstly, the viscosity of the prepared copolymer decreased with the addition of Tung oil due to its plasticizing effect and low viscosity, compared with the viscosity of polyester. Secondly, the prepared copolymer liquid, including polyester base and TO plasticizer, was subjected to curing reaction by addition of peroxide curing agent. During the curing process, a crosslinking reaction occurred at the unsaturation sites in copolymer. This is accompanied by the viscosity increase reaching the gelation step; this is a normal routine regarding the crosslinking of polymers^48^. Further curing takes place that converts the polymer into solid material. This observed change in viscosity is significant because it will be easier to disperse and distribute the desired fillers well in the low-viscous solution in a shorter time before curing; the industrial processing is easier in this case. With the curing reaction, the prepared composites get hardened, taking the applicable improved stable form.

Figure 2(b) describes the minutes taken for the gelation step. It is noticed that the higher content of Tung oil has decreased the gelation time to very fewer minutes. In other words, the copolymer becomes more active toward the curing reaction. The P/TO-50 specimen has a gelation timing of 24 min, compared with 27 min for the neat polymer. The reduced gelation time may be attributed to the abundant unsaturation sites in the natural oil modifier along with the unsaturation sites in the polymer, in addition to the differentiation in the properties of both materials, mainly viscosity, even after mixing together. Basically, the properties of the polymer base are changed during the curing reaction; the liquid resin starts to be crosslinked and converted to the hard form, passing the gelation phase. Actually, reducing gelation time to 24 min in P/TO-50 is a good result, where only 20 min is adequate time appropriate for mixing all fillers in the matrix before the viscosity increases, reaching the gel phase. Thus, the 24 min support and facilitate the facile practical implications for mixing fillers during the preparation phase, especially with the low-viscous P/TO-50 specimen, which doesn’t require longer time for mixing.

After gelation step, the curing continues to take place rapidly alongside the temperature increase. The curing temperature reflects the exothermic property of curing reaction. The crosslinking of unsaturation sites in the resin is accompanied by heat release. It is a good property that the reaction releases lower heat, which is noticed in the proposed composites (Fig. 2(c)). The preparation doesn’t consume energy for heating, as it is an exothermic type. The maximum curing temperatures of P, P/TO-10, P/TO-20, P/TO-30, P/TO-40, and P/TO-50 recorded 130.6 °C, 130 °C, 129.3 °C, 127.7 °C, 126.1 °C, and 125.4 °C, respectively. The observed decrease in curing temperature, particularly in P/TO-40 and P/TO-50 copolymers, indicates lower heat emission during the curing reaction, compared with that of P specimen, by 3.4% and 4%, respectively.

Furthermore, Fig. 2(d) indicates the time taken starting from the gelation step to the maximum curing temperature reached, defined as the gelation-curing period. The oil plasticizer showed a little change in the gelation-curing period, as expected. The effective concentrations are appear to be the P/TO-30, P/TO-40, and P/TO-50 copolymers that alter the gelation-curing timing of P from 7 to 6, 5.1, and 4.7 min, respectively. The Tung oil plasticizer showed a change in the gelation-curing period. It decreased this period regarding blending more contents of the toughening oil containing unsaturation; the new blend has more unsaturation sites available for active curing. Also, the change in this period and the related physical properties presented in all figures infer the successful grafting and curing of composite.

### Exudation test

**Fig. 3:**
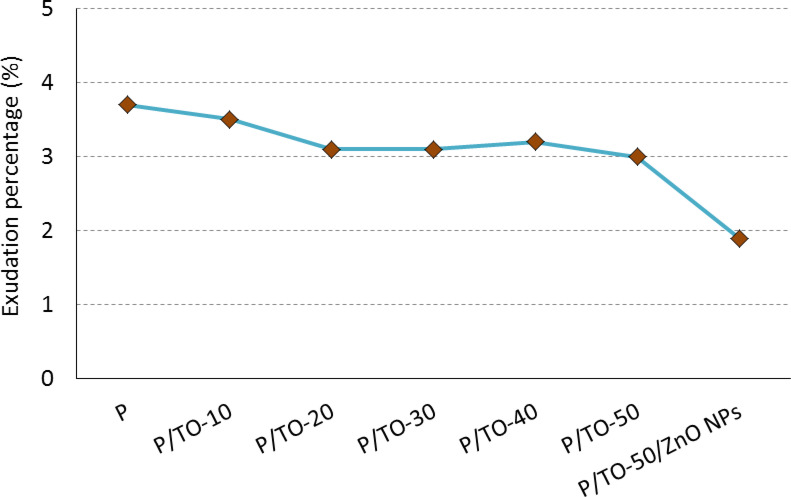
Exudation test for bio-based polyester, Tung oil-based copolymers, and Tung oil-/ZnO NPs-based nanocomposite.

With this test, the stability of composite against exudation is identified. As per Fig. 3, all copolymers have almost the same weight in comparison with P. The absence of weight loss reflects the stability of TO plasticizer in P matrix with concentrations up to P/TO-50. Furthermore, the nanocomposite containing ZnO NPs has the best stability regarding the least percentage of exudation, where the exudation stability was promoted by 48.6%. This improved performance is owing to the progressive filling effect of ZnO NPs as a physical improver. Actually, the nanofiller itself could enhance the physical properties and stabilize the matrix from the environmental conditions, such as exudation, especially in the case of the compatible and well-distributed interface. The P/TO-50/ZnO NPs system sharply decreases the exudation percentage. This scenario matches with that reported elsewhere^49^, where the incorporated high-surface-area nanofillers could decrease the degree of exudation. Besides, all proposed concentrations are more stable than neat P due to the molecular matching. Such data approve the successful grafting of TO and ZnO NPs into the matrix

**Fig. 4:**
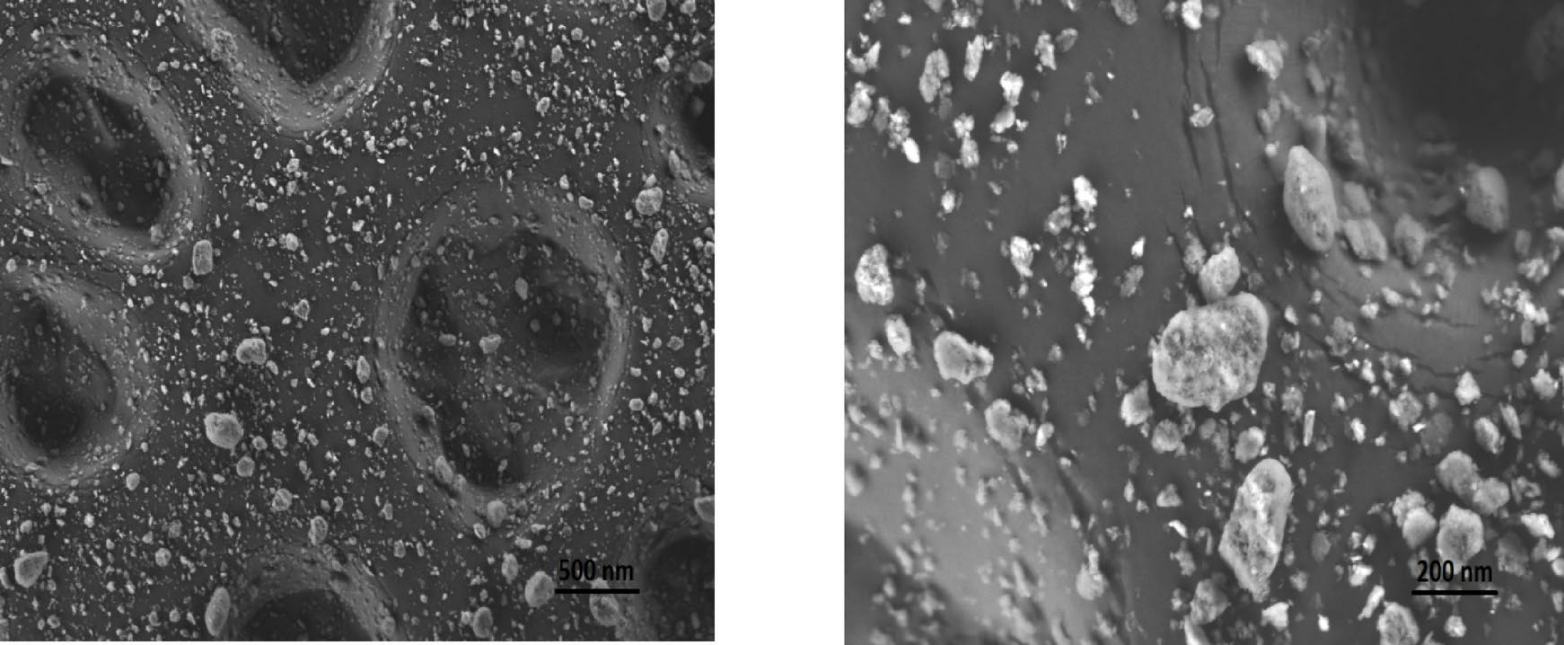
SEM photos of bio-based P/TO-50/ZnO NPs system: low (a) and high (b) magnifications.

For confirming such grafting and dispersion of nanofiller in polymer, the surface morphology is provided. The low and high SEM magnifications of the optimized P/TO-50/ZnO NPs nanocomposite are represented in Figs. 4(a) and (b). It is clear that ZnO NPs are located within the matrix with a distributive property. The filler appears to be spread and grafted over the matrix (exfoliation interface without agglomeration) thanks to the compatibility and good dispersion of fillers in the host polymer upon mechanical stirring and sonication. In other words, the proposed composite system has a filler-grafted surface where fillers and polymer attach to each other effectively. This behavior is clear in the different positions shown in both figures. Basically, TO, as a liquid acid material, was distributed in P polyester matrix liquid phase. Because of the similarity in TO and P natures, their mixture became homogeneous as one phase without separations. This result is noticed by SEM, where the cured polymer provides one phase; the distributed particles screened in morphology stand for the ZnO NPs additive. Such discussion approves the successful grafting and the promoted exudation properties discussed in Fig. 3. As the diffusion of the filling material and plasticizer is established on the contact among all components, the proposed bio-based composites show good intermolecular interaction amongst the polyester matrix, Tung oil, and zinc oxide nanoparticles that provide a stable physical character.

### Creep test

**Fig. 5:**
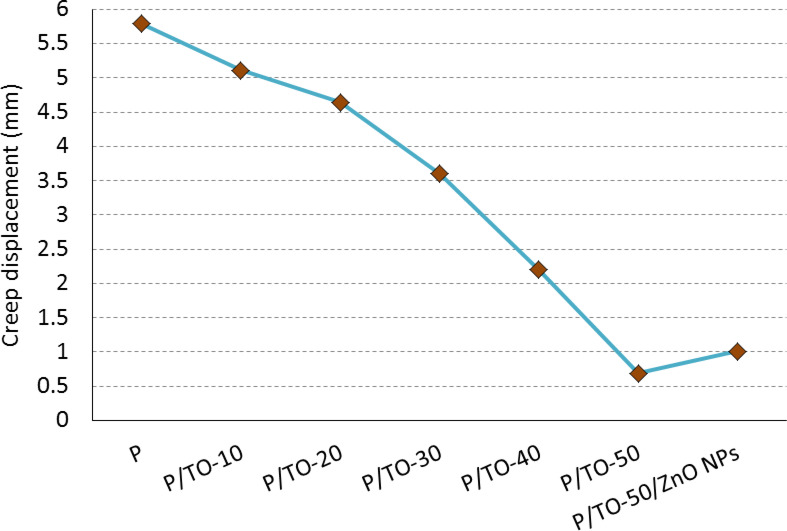
Creep test for bio-based polyester, Tung oil-based copolymers, and Tung oil-/ZnO NPs-based nanocomposite.

The creep measurements for P, plasticized concentrations of P/TO, and P/TO/ZnO NPs nanocomposite are given in Fig. 5. The presented diagram of creep deformation shows the longest creep displacement for the neat polymer (5.79 mm), so more deformation occurred (low creep resistance) compared with the TO- and ZnO NPs-incorporated specimens. The longer creep length reflects the unstable surface of the polymer, which suffers from distortion changes in the polymeric segments. On the other hand, the specimens record shorter creep displacements (high creep resistance) in the case of plasticizing with TO. The more content of TO to be added, the higher the resistance to creep is attained due to the low numbers of creep displacement, as clear in Fig. 5. It is known that the short deformation displacement reflects the stability of the polymer surface^50,51^. Some materials, such as polymers and rubbers, have an elastic nature. When the polymer is subjected to an external force, its segments absorb the force and get stressed. The elastic property lets the stretched segments relax and return to their geometry without failure^52,53^. The elastic material may be a crosslinked one, like crosslinked soft polymers, and this crosslinking may not decline the elasticity nature. Furthermore, the plasticized polymers act as highly elastic materials, as the plasticizer could soften their chains and facilitate their recovery. Consequently, the polymer gives good data-results on the resistance to creep and external forces, especially if it is plasticized as in TO-incorporated specimens. If this recovery force or elastic recovery is able to compensate for the applied force, the polymer shall resist creep, and vice versa. In our case, TO plasticized the taken polymer, so it smoothes and softens the polymer surface. The new surface tries to recover the deformation occurred via the applied creep forces. The resulting deformation (creep displacement) is minimized accordingly, which is highly noticeable with the P/TO-50 copolymer by 88.1%; hence, TO improves creep resistance. The addition of ZnO NPs increased the creep displacement in a minor value due to the stiff nanoparticles; however, it is still lower than neat polymer. The final composite promoted the creep resistance. In other words, TO plasticizer stores the stress applied on P and mends it by returning the mobile/stressed segments to their original positions. Accordingly, the TO avoids the creep deformation and reduces creep displacement. For the practical applications, the proposed plasticized composites are beneficial for boosting the overall performance of bio-based copolymers based on compensating for the creep displacement in addition to the improved flexural behavior. Overall, the proposed modifying materials offer an entanglement to the polymer segments and control the deformation of the overall composite.

### Impression test

**Fig. 6:**
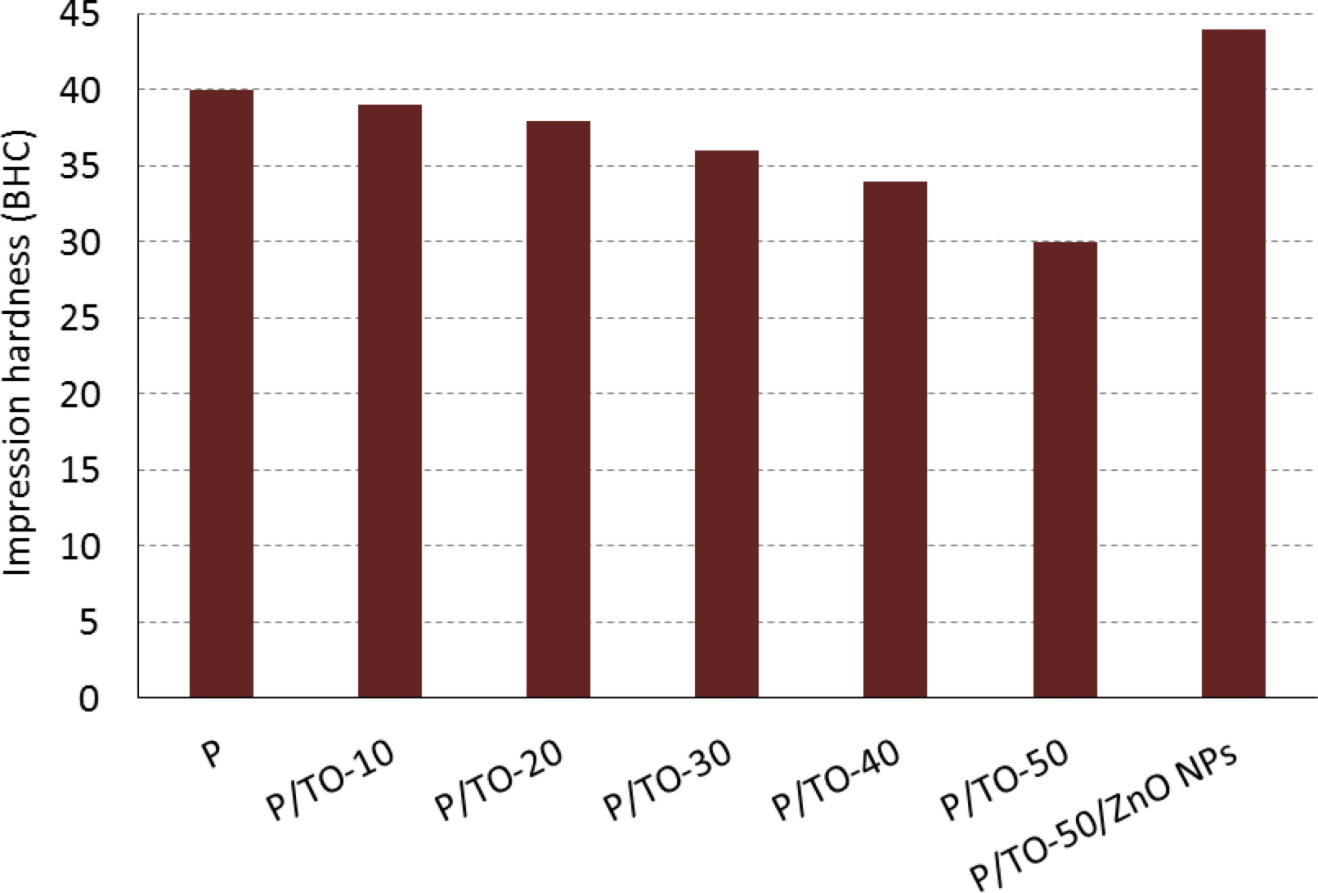
Impression hardness of bio-based polyester, Tung oil-based copolymers, and Tung oil-/ZnO NPs-based nanocomposite.

The impression test was performed to neat P, P/TO copolymers, and P/TO/ZnO NPs nanocomposite to investigate the effectiveness of TO and ZnO NPs on the hardness. Firstly, the TO-based specimens with low concentrations have a minor effect on hardness with a small reduction in the hardness values, as provided in Fig. 6. Adding more concentrations of TO results in plasticizing the polymer segments and altering their mechanical properties. This behavior demonstrates the specimen’s capacity to tolerate imposed hardness^54^. The highest concentration (P/TO-50) caused a reduction in hardness by 25%. However, this concentration was promoted by the effect of ZnO NPs, where the hardness value of P/TO-50/ZnO NPs nanocomposite increased by 46.7%, compared with the P/TO-50 specimen. The hardness of P/TO-50/ZnO NPs nanocomposite exceeded that of the blank P as well. From the results, there was an initial decrease in hardness values due to the plasticizing effect of TO plasticizer (induced softness). On the other hand, the addition of ZnO NPs led to subsequent improvement in hardness due to the effect of stiff nanofiller, so the surface became harder and could resist the applied force. The nanofiller itself is capable of developing the hardness of the different matrices in general; also, the filler distribution and direction control the resulted enhancement^55^. Overall, ZnO NPs reinforce and stabilize the plasticized copolymer matrix by increasing its impressive hardness, even after adding the TO plasticizer.

### Flexural strength

**Fig. 7:**
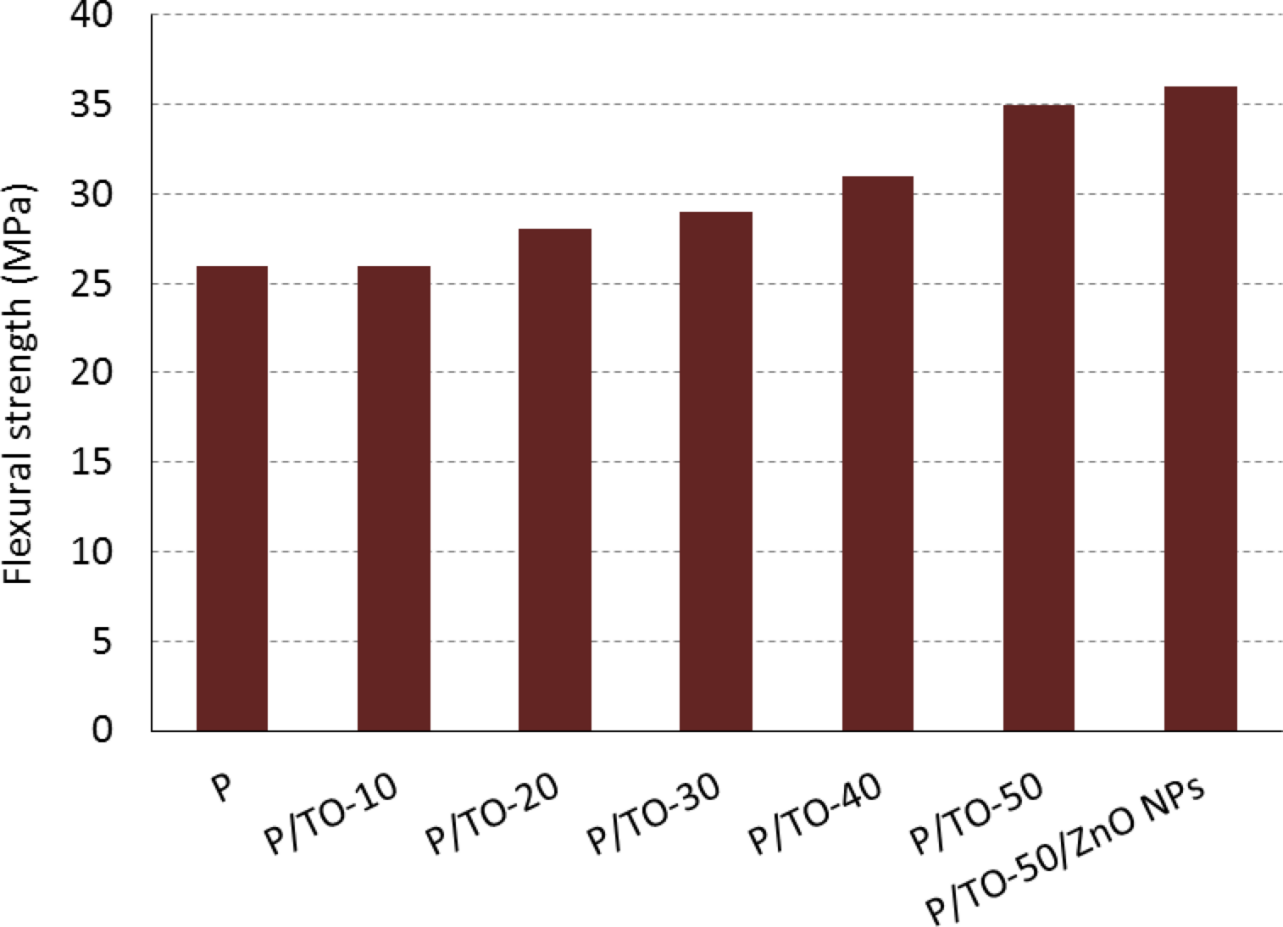
Flexural strength of bio-based polyester, Tung oil-based copolymers, and Tung oil-/ZnO NPs-based nanocomposite.

The effect of TO plasticizer and ZnO NPs on the flexural strength of P polymer is investigated; the results are illustrated in Fig. 7. The P, P/TO-10, P/TO-20, P/TO-30, P/TO-40, and P/TO-50 specimens recorded flexural strength at 26 MPa, 26 MPa, 28 MPa, 29 MPa, 31 MPa, and 35 MPa, respectively. It is clear that the higher the content of TO added, the more it positively affects the flexural properties. The obtained specimens have flexible behavior, where the plasticizing additive makes the polymer tougher. This is noticed especially with the P/TO-50 concentration, which enhanced the flexural strength by 34.6%, compared with P. The flexural strength was increased further by the second addition of ZnO NPs, as it reads 36 MPa. TO and ZnO NPs caused improvement in the flexural strength, where they made the polymer capable of absorbing more loading without failure. The composites seem to bear additional flexural force (more flexible design), compared with neat polymer. Furthermore, the Tung oil/ZnO-containing specimen has additional properties, such as hardness and stiffness, along with the flexibility. Thus, this added-value hybrid composite can serve as an optimized formula for both flexible and durable applications. In conclusion, both TO and ZnO NPs fillers developed the flexural strength of the proposed bio-based polymer, resulting in toughening the overall composite.

### Thermal properties

**Fig. 8:**
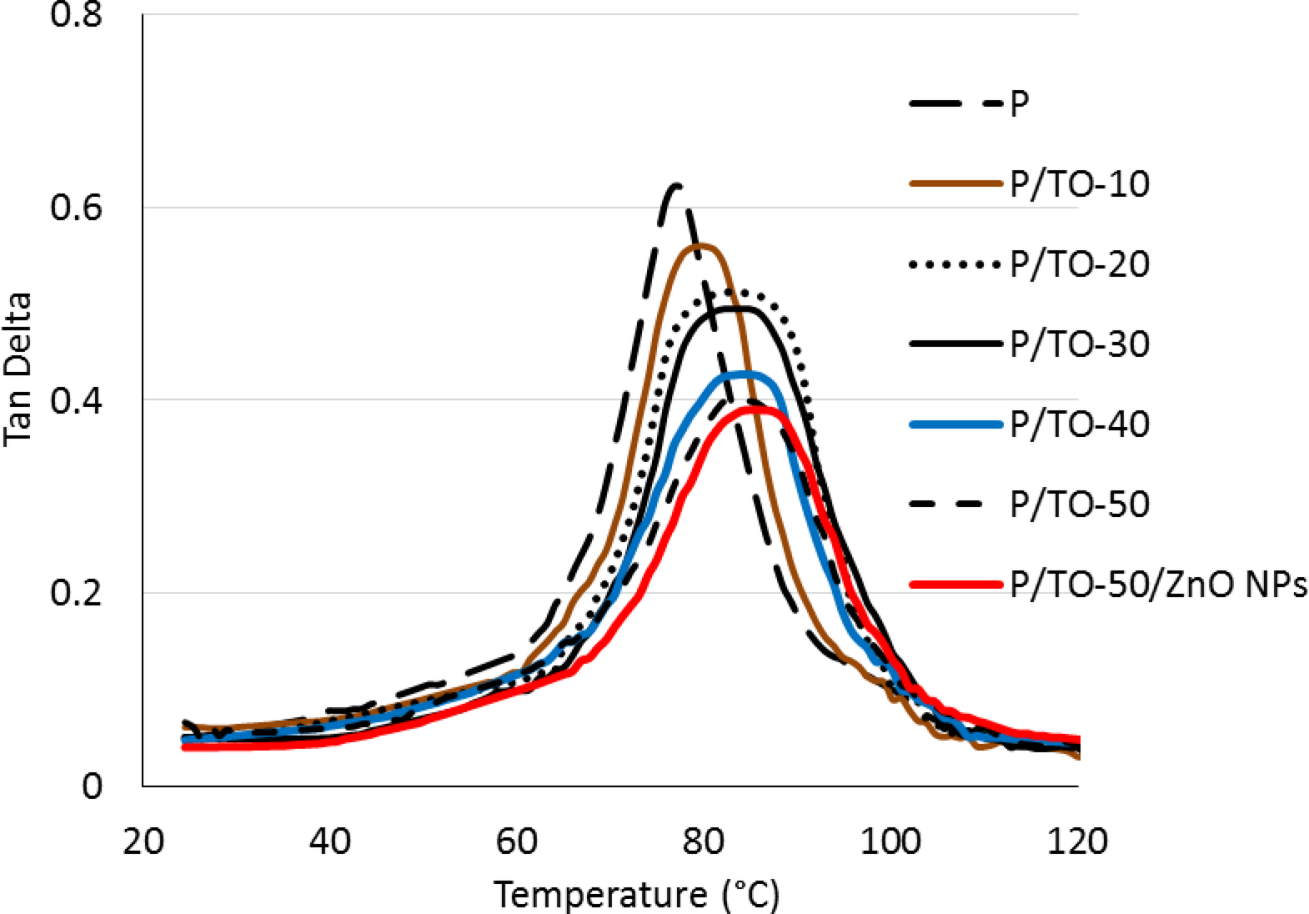
Tan Delta-temperature plateaus for bio-based polyester, Tung oil-based copolymers, and Tung oil-/ZnO NPs-based nanocomposite.

The effect of TO and ZnO NPs materials on the thermal properties of used P polyester is investigated by dynamical mechanical analysis. The values of tan Delta and the glass transition temperature (T_*g*_), as a function of the testing temperatures, are determined. The tan Delta is a value that is calculated by the DMA software during testing. It is the quotient of loss factor by modulus. From the tan Delta-temperature curve, the corresponding T_*g*_and damping peak parameters are identified for studying the thermal characteristics^56^. The tan Delta-temperature plateaus, including values of tan Delta peaks and T_*g*_ temperatures, are represented in Fig. 8. As the T_*g*_ is the temperature at which the polymer chains can move freely with more molecular motions, the T_*g*_ of polymers and polymer composites is identified as the temperature located at the peak of tan Delta-temperature curve, where this peak elucidates the highest fraction of viscous response, compared with elastic response, with increasing the free volume under the testing conditions of temperature increase. In other words, as tan delta numbers are the values between loss and storage modulus, their peak represents the point between the glassy and rubbery phases. Identification of T_*g*_ from Tan delta curve is a new technique. Hence, the transition between the hard glassy phase and the rubbery phase (tan Delta peak) of the taken polymer is expressed as its T_*g*_^57,58^. The thermal behavior of P and all specimens shows similar scenarios regarding two different phases: glassy (with restricted mobility) and rubbery (viscous). The phases exist in the ranges of room temperature and ~ 75 °C, and from ~ 85 °C to ~ 110 °C, respectively. The obtained peaks are the transitions present between these phases. It is clear that the highest peak belongs to the neat P polymer. Furthermore, adding more concentrations of TO results in dropping the peaks in the curves of tan Delta-temperature. The low number of damping factor is based on the more flexural properties measured, so the proposed concentrations show reduced peaks dramatically. The P/TO-50 formula has the least value of tan Delta (0.4) through a lowering percentage of 35.5%, compared with P. In addition, the P/TO-50/ZnO NPs nanocomposite has a low tan Delta-temperature peak. Alteration of peaks in damping curves is well-known owing to the progressive effect of filling components on the associated viscoelastic properties^59^. The accurate values of tan Delta peaks and T_*g*_ temperatures are identified separately in Table 1 for more clarification, as these numbers are not presented clearly in the large scale of temperatures present in the whole tan Delta plateaus. For the T_*g*_ measurements, the addition of more concentrations of TO is accompanied by shifting the screened temperatures to higher ones. This is realized dramatically by P/TO-10, P/TO-20, P/TO-30, P/TO-40, and P/TO-50 specimens that achieved T_*g*_ at 77.6 °C, 81.2 °C, 82.1 °C, 82.5 °C, and 83 °C, respectively, compared with 76.7 °C for neat P. The functional unsaturation sites existing in TO plasticizer are expected to share with the curing reaction in conjunction with unsaturation sites in P polyester, where the polymerization takes place via a dual system with heavy crosslinking sites. Some enhancement in T_*g*_ values is observed. Furthermore, the P/TO-50/ZnO NPs nanocomposite has increased T_*g*_ temperature by ~ 10%, due to the diminution of free space and segmental mobility. The proposed fillers, especially ZnO, delay the softening of polymeric chains during heating; as for the mechanism of nanomaterials, the T_*g*_ values increase accordingly. The existing data reveal the successful blending of TO and ZnO NPs into the bio-based polyester that improves its glass transition temperature and thermal properties.

**Table 1:** Values of Tan Delta peaks, and T_*g*_ temperatures of bio-based polyester, Tung oil-based copolymers, and Tung oil-/ZnO NPs-based nanocomposite.

Specimen	tan Delta peak (value)	T_g_(°C)
P	0.62	76.7
P/TO-10	0.56	77.6
P/TO-20	0.51	81.2
P/TO-30	0.49	82.1
P/TO-40	0.43	82.5
P/TO-50	0.40	83
P/TO-50/ZnO NPs	0.39	84.3

From the previous data, it is aimed to promote the properties of environmentally friendly plasticized polymer after adding different concentrations of TO plasticizer. In fact, the improved P/TO-50 copolymer still has some drawbacks, such as low strength, although it is tougher and more elastic and has higher thermal stability and better physical properties. The 3% ZnO NPs concentration was selected based on the literature on using NPs for enhancement of composites; different concentrations above 3% were found to form aggregated and heterogeneous structures with inferior characteristics^60,61^. So, the 3% ZnO NPs concentration was chosen to adjust the preferred P/TO-50 formulation to improve other properties such as hardness. The proposed hybrid was then taken as an optimized composite that possesses superior characteristics.

### Statistical analyssis

**Fig. 9:**
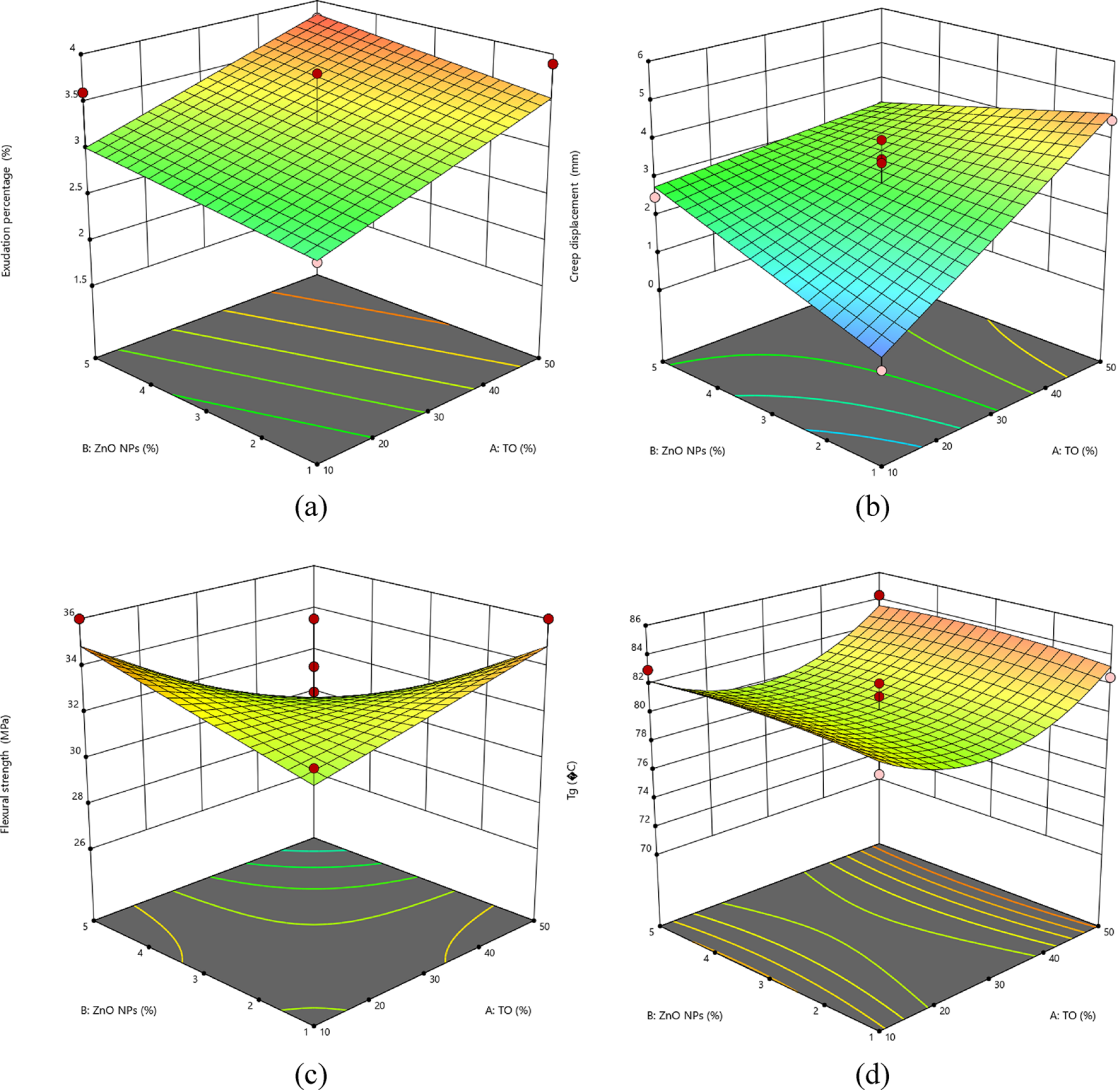
RSM statistical analysis of Tung oil and ZnO NPs main parameters and the related exudation percentage (a), creep displacement (b), flexural strength (c), and T_*g*_ (d) responses.

The response surface methodology (RSM) statistical analysis was performed to compare the statistical difference between the treatments more effectively. The models were performed by ANOVA software. The considered coefficient of determination (R^2^) is ranged between 73 and 95, which supports the adequacy of recognized models with empirical data^62,63^. Furthermore, the most important responses of exudation, creep, flexural, and T_*g*_, based on the main factors, are represented in Fig. 9. The 3D response surface plots demonstrate that TO and ZnO NPs main parameters control the properties. The more recommended levels are optimized by the TO 50% and ZnO NPs 3% treating factors as the highest efficient inputs. These results demonstrate that the model fits the empirical data effectively. The interaction between inputs and the resulting influence on the responses supports the thermal and physical stability and the improved mechanical profile, as per what was noticed in the results and characteristics provided before.

## Conclusion

In this paper, natural Tung oil (TO) and zinc oxide nanoparticles (ZnO NPs) were utilized to modify the physical and mechanical properties of bio-based polyester (P). The proposed copolymers were formulated successfully based on P/TO-10, P/TO-20, P/TO-30, P/TO-40, and P/TO-50 by wt%. The P/TO-50 copolymer was filled with 3 wt% ZnO NPs to enhance its characteristics. Due to the plasticizing effect of TO, it was observed that adding TO reduced viscosity; the lowest viscosity was seen at greater concentrations. The higher content of TO, particularly with P/TO-40 and P/TO-50 copolymers, shortened the gelation time and curing temperature. In addition, the P/TO-30, P/TO-40, and P/TO-50 copolymers showed shorter gelation-curing periods regarding blending more natural toughening oil. The evaluation of exudation properties assured the stability of all TO-incorporated copolymers in comparison with P. The P/TO-50/ZnO NPs nanocomposite achieved more stability with a lower exudation percentage due to grafting and distribution of ZnO NPs, as detected by SEM. Mechanically, the creep data showed the longest displacement in neat P, compared with the TO-based specimens. Also, the proposed P/TO-50/ZnO NPs system displays an improved profile. For hardness, the concentrations of TO caused a reduction in hardness; however, adding ZnO NPs improved the related value. In addition, both TO and ZnO NPs promoted the flexural strength of P; this reflects the flexural behavior of the plasticizing filler. In the presence of TO and ZnO NPs, the brittle crosslinked polymer becomes toughened through the elastic recovery advantage. The gained features enhance the material’s ability to keep the polymer as a reinforced enhanced product that withstands external mechanical forces without failure or deformation. Thermally, the neat P, P/TO copolymers, and ZnO NPs-based nanocomposite show two different phases of glassy and rubbery regions. The highest peak in tan Delta-temperature curves belongs to the neat P polymer. However, grafting TO altered the peak due to the more flexural property in proposed composites. Furthermore, it was found that T_*g*_ was enhanced by including more concentrations of TO and ZnO NPs fillers compared with neat P. The results of statistical analysis demonstrate the interaction between inputs and ensuing responses as detected by different testings. The proposed additives support P matrix with stability against applied mechanical forces and physical conditions via a new paradigm to design bio-based composites for potential applications.

## Data Availability

All data generated or analysed during this study are included in this published article.
